# Genome-wide characterization of aldehyde dehydrogenase gene family members in groundnut (*Arachis hypogaea*) and the analysis under saline-alkali stress

**DOI:** 10.3389/fpls.2023.1097001

**Published:** 2023-02-16

**Authors:** Xiaoming Zhang, Jingwen Zhong, Liang Cao, Chunyuan Ren, Gaobo Yu, Yanhua Gu, Jingwen Ruan, Siqi Zhao, Lei Wang, Haishun Ru, Lili Cheng, Qi Wang, Yuxian Zhang

**Affiliations:** ^1^ Heilongjiang Bayi Agricultural University, Key Laboratory of Soybean Mechanized Production, Ministry of Agriculture and Rural Affairs, Daqing, China; ^2^ Agricultural College, Northeast Agricultural University, Harbin, China; ^3^ National Coarse Cereals Engineering Research Center, Heilongjiang Bayi Agricultural University, Daqing, China; ^4^ Institute of Industrial Crops, Heilongjiang Academy of Agricultural Sciences, Harbin, China; ^5^ Institute of Crop Cultivation and Tillage, Heilongjiang Academy of Agricultural Sciences, Harbin, China

**Keywords:** aldehyde dehydrogenase, evolutionary, *cis*-acting elements, expression pattern, saline-alkali stress

## Abstract

Groundnut or peanut (*Arachis hypogaea*) is a legume crop. Its seeds are rich in protein and oil. Aldehyde dehydrogenase (ALDH, EC: 1.2.1.3) is an important enzyme involved in detoxification of aldehyde and cellular reactive oxygen species, as well as in attenuation of lipid peroxidation-meditated cellular toxicity under stress conditions. However, few studies have been identified and analyzed about ALDH members in *Arachis hypogaea*. In the present study, 71 members of the ALDH superfamily (AhALDH) were identified using the reference genome obtained from the Phytozome database. A systematic analysis of the evolutionary relationship, motif, gene structure, *cis*-acting elements, collinearity, Gene Ontology (GO) and Kyoto Encyclopedia of Genes and Genomes (KEGG) enrichment, and expression patterns was conducted to understand the structure and function of *AhALDH*s. *AhALDH*s exhibited tissue-specific expression, and quantitative real-time PCR identified significant differences in the expression levels of *AhALDH* members under saline-alkali stress. The results revealed that some *AhALDHs* members could be involved in response to abiotic stress. Our findings on *AhALDHs* provide insights for further study.

## 1 Introduction

Aldehyde molecules are essential intermediate compounds generated in catabolic and biosynthetic pathways during biological development and growth (Vasiliou et al., 2000). In response to stress, aldehyde accumulates in cells, causing an imbalance and interfering with cellular homeostatic metabolic responses (Bartels, 2001), and becomes toxic if present in excess (Carmona-Molero et al., 2021) Aldehyde dehydrogenase (ALDH, EC: 1.2.1.3) as a scavenger of aldehyde molecules contributes to their homeostasis (Yoshida et al., 1998). The ALDH family is composed of a variety of NAD(P)^+^-dependent enzymes that irreversibly oxidize endogenously and exogenously derived aldehyde molecules to carboxylic acids (Yoshiba et al., 1997). ALDH enzymes also function in intermediary metabolism by providing protection from osmotic stress and generating NAD(P)H (Kelly and Gibbs, 1973; Ishitani et al., 1995; Brocker et al., 2010). ALDHs have been reported to improve stress resistance in crops (Bartels and Sunkar, 2005). ALDH family members are found in prokaryotic and eukaryotic organisms and are highly conserved and well represented in virtually all plant species (Brocker et al., 2013). Research on the ALDH gene family in prokaryotes and mammals is abundant (Kirch et al., 2004). The ALDH members associated with ALDH enzyme activity are linked to diseases such as cataracts, hyperprolinaemia, and cancers (Jackson et al., 2011). However, the functional and structural characterization of plant ALDHs and gene duplication events underlying their diversification have lagged behind that of their mammalian and bacterial counterparts (Zhang et al., 2012).

ALDH family members, which are found in almost all plant species, are variable, widespread in plant tissues, and regulated developmentally (Chen C, et al., 2014; Guo et al., 2017). ALDH members participate in plant growth and development and play a vital role in catabolic and bio-synthetic pathways, such as carnitine biosynthesis (Marchitti et al., 2008), glycolysis/gluconeogenesis (Tylichová et al., 2010), and amino-acid metabolism (Yang et al., 2011). The *ALDH2B2* (*rf2*) gene has ALDH domain functions and is a male fertility restorer in maize (Skibbe et al., 2002). *ALDH7s* in *Arabidopsis* and soybean are involved in aldehyde detoxification (Shin et al., 2009), whereas *OsALDH7* is essential for seed maturation, and its mutation leads to seed browning during seed drying and storage of rice (Shin et al., 2009). An increasing number of studies have shown that some ALDH members indirectly function in plant cell protection under various abiotic stresses through detoxification of cellular reactive oxygen species (ROS) and/or reduction of lipid peroxidation (Shin et al., 2009; Singh et al., 2013). Betaine aldehyde dehydrogenases (BADH) are a type of ALDH10 enzyme that catalyze the oxidation of betaine aldehyde into glycine betaine (a major cellular osmolyte) and thereby improve plant resistance to environmental stress (Le Rudulier et al., 1984); the *BADH* gene has been shown to improve salt tolerance in plants (Zhou et al., 2008). Furthermore, the ectopic expression of *ALDH3I1* and *ALDH7B4* significantly reduced malondialdehyde (MDA) levels and lipid peroxidation in transgenic *Arabidopsis*, revealing the role of these two genes in increasing plant tolerance to drought and salt stress (Kotchoni et al., 2006). *VvALDH2B4*, an ALDH member from the Chinese wild grapevine (*Vitis pseudoreticulata*), lowered MDA levels and enhanced plant resistance to salt stress and pathogenic bacteria in over-expressed transgenic *Arabidopsis* (Wen et al., 2012). *ScALDH21*, an ALDH member isolated from *Syntrichia caninervis*, enhanced the activity of antioxidant enzymes, increased the proline content, and lowered the MDA content in transgenic tobacco under salt and drought stress, which is likely the reason for increased germination ratios and root lengths in tobacco plants (Yang et al., 2015).

ALDH members have been identified in *Arabidopsis* (Kirch et al., 2001), rice (*Oryza sativa*) (Gao and Han, 2009), maize (*Zea mays*) (Jimenez-Lopez et al., 2010), soybean (*Glycine max*) (Kotchoni et al., 2012), and cotton (*Gossypium hirsutum*) (Guo et al., 2017), but few studies have examined their presence in groundnut (*Arachis hypogaea*). Groundnut (peanut), a member of the legume family, originated in southern Bolivia (Krapovickas and Gregory, 1994). It is cultivated in more than 100 countries on 26 million hectares (ha) of land for its seeds that are a rich source of dietary fiber, minerals, vitamins, and bioactive compounds, especially proteins and oil (Desmae et al., 2019). The growth of groundnut is affected by abiotic stresses, and research has been conducted to increase its stress resistance through molecular breeding (Desmae et al., 2019). In the present study, ALDH members in groundnut were identified, and a comprehensive analysis (location, evolution, motif, gene structure, *cis*-acting elements, and expression patterns) was conducted. The results can be utilized to breed groundnut with improved stress resistance.

## 2 Materials and methods

### 2.1 ALDH members of *Arachis hypogaea*

The reference genome (*Arachis hypogaea* v1.0) and adjoining information (protein sequence, mRNA, coding sequence, and DNA) were obtained from the Phytozome database (Goodstein et al., 2012). The ALDH domain (accession number PF00171) was downloaded from the PFAM database. The ALDH members were identified through hmmsearch and hmmbuild using the perl script in the Linux system, in which 1e^−15^ was set as a filter threshold (Zhang et al., 2020). The SMART software (Letunic and Bork, 2018) was used to confirm the ALDH domain and remove duplicates.

### 2.2 Analysis of ALDH members

The evolutionary relationship of ALDH members was analyzed using neighbor-joining methods and a Poisson model implemented in MEGA X, with 1000 bootstrap repetitions (Kumar et al., 2018). The motif of ALDH members was analyzed using the MEME platform, in which the length of motifs was 10–15 amino acids, while the e-value of motifs was less than e^−5^ (Bailey et al., 2009). The gene structure of the ALDH members was analyzed using the GSDS software (Hu et al., 2015). The *cis*-acting elements were identified and predicted using PlantCare, in which the function of each *cis*-acting element was predicted (Lescot et al., 2002). The expression of ALDH members and their location on chromosomes was extracted from the Phytozome database (Goodstein et al., 2012). The results were plotted using TBtool (Chen et al., 2020). The Gene Ontology (GO) and Kyoto Encyclopedia of Genes and Genomes (KEGG) databases were used for the gene annotation of ALDH members (Chen et al., 2017); Majorbio Cloud provided the platform for enrichment analysis (Shanghai, China).

### 2.3 Plant materials and conditions

The locally grown groundnut cultivar Silihong was used in this study. The seeds were provided by the Institute of Economic Botany, Heilongjiang Academy of Agricultural Sciences (Harbin, Hei Longjiang, China). Seeds of the same size were sterilized with 5% NaClO solution for 15 min, rinsed with distilled water, and placed in a Petri dish lined with double-layer filter paper. The seeds were covered with a single filter paper. Three replicates of 10 seeds per Petri dish were prepared for saline-alkali and control treatments. Petri dishes were placed in a GXZ intelligent light incubator (Ningbo Jiangnan Instrument Factory, Zehjiang, China) at 25°C in the dark. When the length of the peanut buds reached half the length of the peanut seeds, the seeds were sampled and used for expression pattern analysis. Control seeds were treated with distilled water. The rest of the seeds were subjected to saline-alkali treatment by adding saline-alkali solution (pH, 8.9) to Petri dishes. The sprouts in each Petri dish were set as the experimental unit, while each treatment had three experimental units as biological replicates.

### 2.4 Physiological and expression level analysis

The radicle was used as samples in this study, with biological and technological replicates. ALDH enzyme activity was determined under water and saline-alkali treatment at 0, 12, 24, 48, 72, and 96 h using a Multiskan FC microplate reader (Thermo Scientific Company, Wilmington, DE, USA) and the Elisa Kit (M0608, Michy Biology, Suzhou, China) according to the manufacturer’s instructions. Radicles were used as samples. The samples at 0 and 48 h from both treatments were used for quantitative real-time PCR (qRT-PCR) analysis. Total RNA was extracted using a MolPure^®^ Plant Plus RNA Kit (19292ES, Yeason, Shanghai, China). The qualified RNA was used for reverse transcription using a kit (11139ES, Yeason) after detection using a Nanodrop OneC (Thermo Fisher Scientific, Waltham, MA, USA) and 1% agarose for RNA quality. The primers for ALDH amplification were designed using DNAMAN (Lynnon Biosoft, San Ramon, CA, USA) (Nong et al., 2019), and the *UKN1* gene was used as the reference gene (**Table S2**). qRT-PCR was performed on the Roche platform (480 II, Roche, Basel, Switzerland) using Hieff UNICON^®^ Universal Blue qPCR SYBR Green Master Mix (11184ES, Yeason) according to the manufacturer’s instructions. The expression levels of ALDH members were calculated using the 2^−ΔCt^ method (Livak and Schmittgen, 2001). Data were analyzed in the SPSS software (Bezzaouha et al., 2020). Duncan’s multiple method was used to test differences between groups.

## 3 Results

### 3.1 Identification and evolution of ALDH members

**Figure 1:**
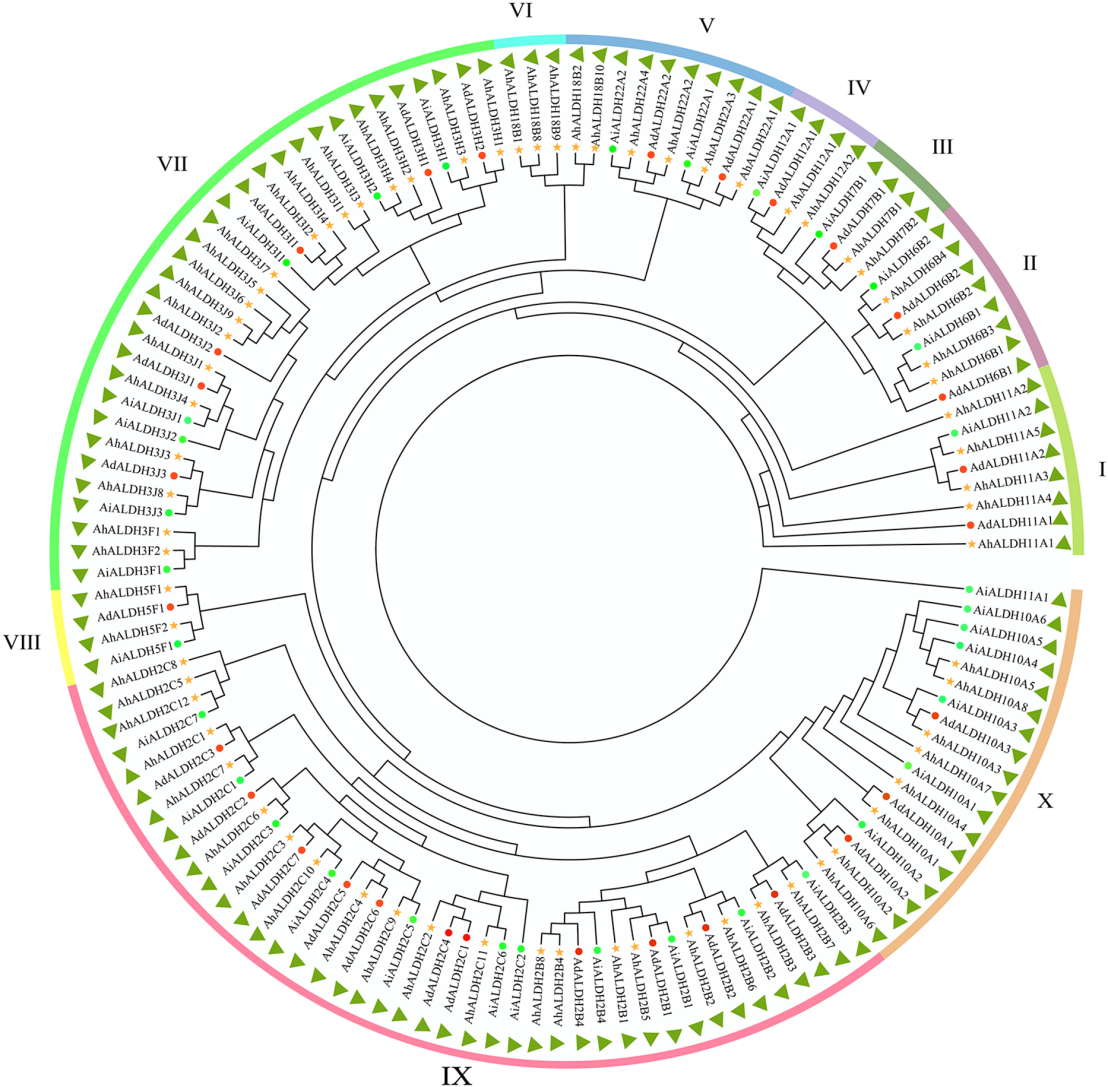
Evolutionary relationship of AhALDH members. Green circles represent ALDH members in *Arachis hypogaea*, while red circles and yellow stars represent ALDH members in *Arachis duranensis* and *Arachis ipaensis.* Outer ring bands in different colors represent the subfamilies I–X.

One hundred and five ALDH members were identified from the reference genome (*Arachis hypogaea* v1.0) from the Phytozome database. The sequences were confirmed using the Smart software (Letunic and Bork, 2018), and after removing the duplicates, 71 members of the ALDH family were identified. The evolutionary relationships of the AhALDH members were analyzed using the MEGA X software (Kumar et al., 2018), while ALDH members in *Arachis duranensis* and *Arachis ipaensis* were used to correct evolutionary relationships. All these ALDH members were classified into 10 subfamilies, labelled I–X according to the order of AhALDH evolution. Subfamilies III and VI had the fewest AhALDH members, only two, while subfamily X had the highest number of AhALDH members at 17. These AhALDH members were named according to the evolutionary results and Brockers’ method (Brocker et al., 2013). ALDH members in *A. duranensis* and *A. ipaensis*, along with AhALDH members, were also divided into 10 subfamilies, which proved the accuracy of the evolutionary analysis (**Figure 1**). Moreover, detailed information about the proteins (including protein length, molecular weight, and isoelectric point) is shown in **Table S1**. The protein length varied from 303 to 791 amino acids, the molecular weight was 32.48–88.83 kDa, and theisoelectric point was 4.91–9.61. This information revealed that AhALDH members in different subgroups had diversified features, indicating that different members might perform different functions.

### 3.2 Location analysis of AhALDHs

**Figure 2:**
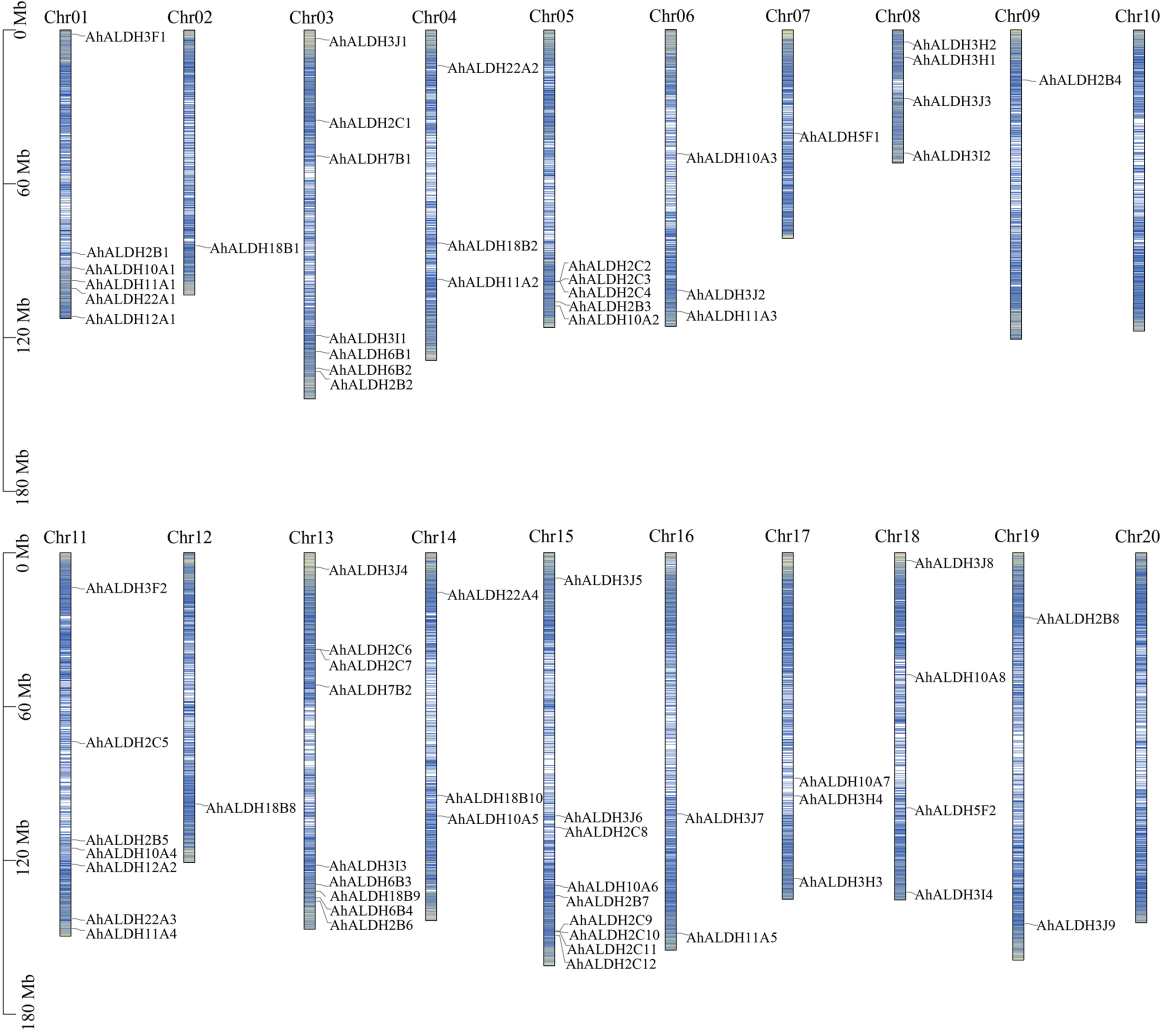
Location of *AhALDH* members on chromosomes (Chr1–Chr20). The length of the columns represents the size of the chromosomes. The black scale on the left represents the position, and blue lines inside the columns represent the gene density on chromosomes.

Location information was obtained from databases and drawn using Tbtools (**Figure 2**). All chromosomes had AhALDH members, except for Chr 10 and Chr 20. The number of AhALDH members on each Chr differed: Chr13 and Chr15 had the most AhALDH members (9), while Chr03 and Chr11 had seven members; Chr1, Chr7, and Chr12, with only one member each, had the fewest, while only two AhALDH members were found on Chr16 and Chr19. These results indicated that the distribution of AhALDH members on groundnut chromosomes is not uniform, indicating that Chr13 and Chr15 might be central to AhALDH evolution.

### 3.3 Motif and gene structure analysis of AhALDHs

**Figure 3:**
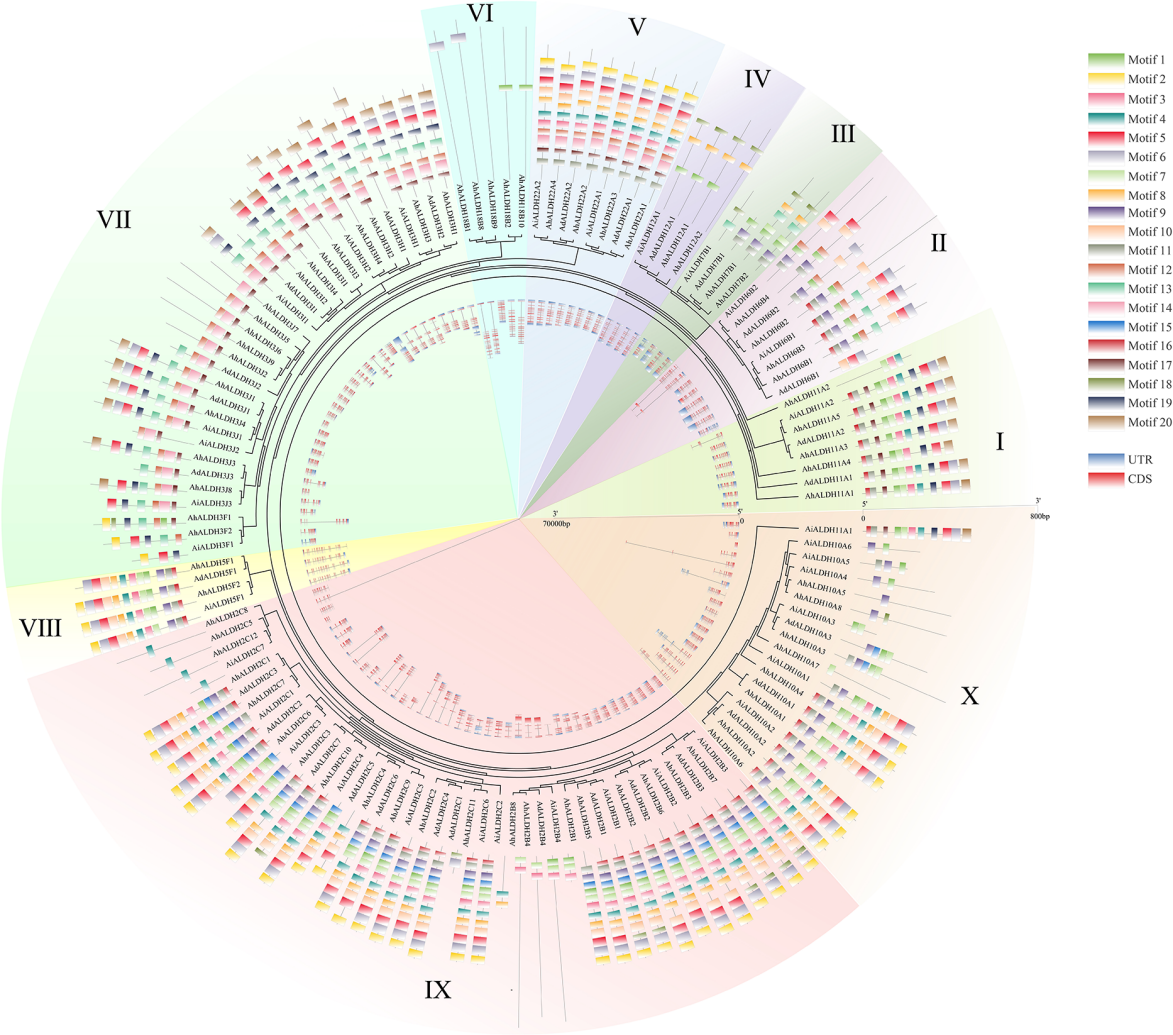
Motif and gene structure analysis of AhALDH members. The middle ring represents the evolutionary relationship of AhALDHs. The 10 families are highlighted with differently colored backgrounds. The outer ring shows the motif analysis of AhALDHs. Motifs from 1 to 20 are marked with differently colored boxes. The inner ring displays the gene structure analysis of AhALDHs. Blue boxes are untranslated regions (UTRs) and pink boxes are coding sequences (CDSs), black lines are the intron region.

The motif and gene structure of AhALDHs were analyzed using MEME and GSDS, respectively, to explore their structure and properties. The results are shown in **Figure 3**. The length of the motifs varied from 10 to 50, and the e-value was lower than 1e^−20^. AhALDHs in the same subfamily shared similar kinds of motifs, although some motifs also varied within the same subfamily, such as motif 1 (**Figure 3**). Members of the same subfamily had a similar gene structure, with the longest AhALDH member being in subfamily X. Moreover, the motif and gene structure of ALDH members in *A. duranensis* and *A. ipaensis* also revealed that members in each subfamily had similar characteristics, indicating that they might perform similar functions.

### 3.4 Comparison of ALDHs in different species

**Figure 4:**
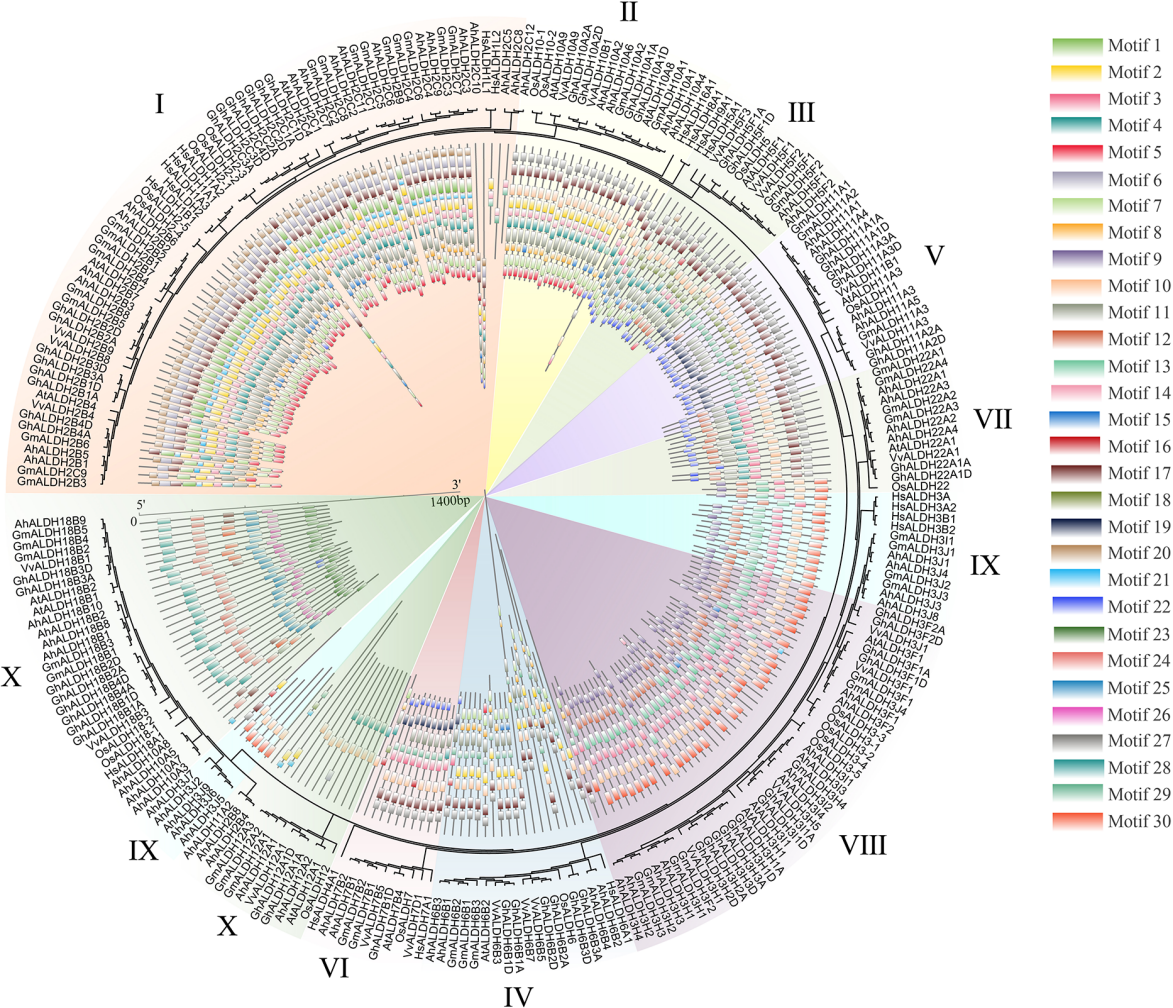
Analysis of ALDH members in seven species. The seven species were groundnut (*Ah*), *Arabidopsis* (*At*), rice (*Os*), human (*Hs*), cotton (*Gh*), soybean (*Gm*), and grape (*Vv*). Differently colored backgrounds represent different subfamilies. The inner circle represents the motifs of ALDH members; different squares show different motifs.

In this study, 260 ALDH members in seven species, namely groundnut, *Arabidopsis*, rice, human, cotton, soybean, and grape (*Vitis vinifera*), were used for the evolutionary and motif analysis. These 260 members were divided into 10 subfamilies, in which the members within each subfamily shared similar motifs. The evolutionary results were similar to those shown in **Figure 2**. Under the same analysis threshold, the AhALDHs had similar kinds of motifs to ALDH members in other species (**Figure 4**).

### 3.5 Cis-acting element analysis of AhALDHs

**Figure 5:**
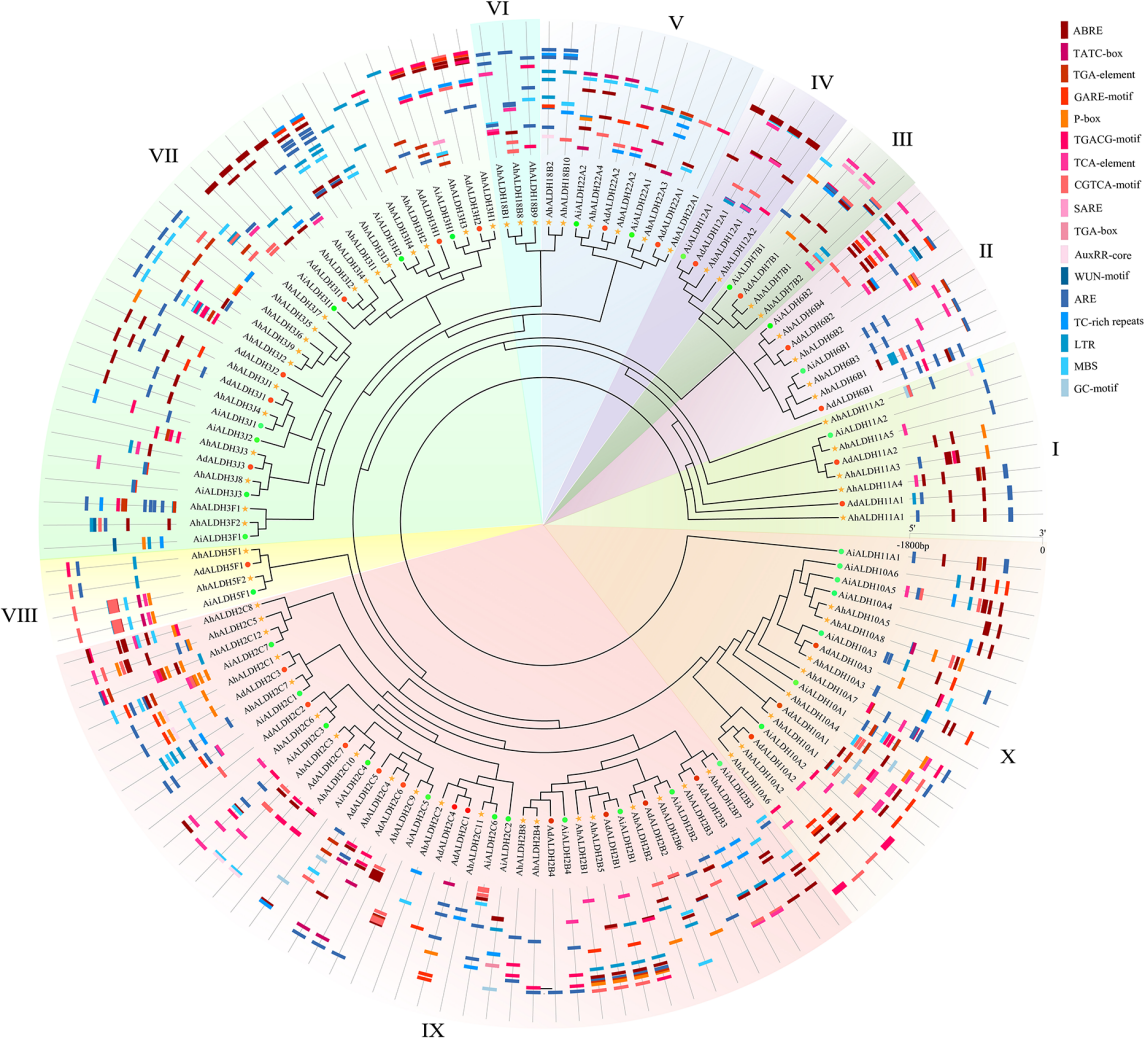
*Cis*-acting element analysis of *AhALDH* members. The inner ring shows the evolutionary relationship of AhALDHs; differently colored backgrounds represent different subfamilies. The outer ring shows the *Cis*-acting elements analysis. Red, orange, and pink boxes in different shapes represent *cis*-acting elements associated with hormones, while the blue boxes are *cis*-acting elements responding to abiotic stress.

The *cis*-acting elements of *AhALDH*s were analyzed and their function predicted using the Plantcare software (**Figure 5**; **Table S3**). Three kinds of elements were recognized based on their function. The *cis*-acting elements TGA-element, ABRE, P-box, GARE-motif, TCA-element, AT-rich sequence, SARE, TATC-box, and AuxRR-core (marked in red in **Figure 4**) were predicted to be related to hormones; LTR, MBS, ARE, and GC-motif (marked in blue) were related to abiotic stress. The members within each subfamily had similar *cis*-acting elements, and the analysis revealed that *AhALDH*s might be associated with hormones and abiotic stress; in particular, some members had stress-related *cis*-acting elements.

### 3.6 Collinearity analysis of AhALDHs

**Figure 6:**
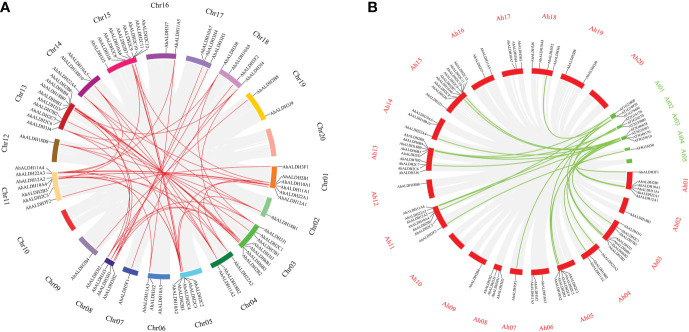
Collinear analysis of *AhALDH* members in groundnut and *Arabidopsis*. **(A)** Collinear analysis of *AhALDH* members in *Arachis hypogaea.* The circles of different colors represent different chromosomes, while red lines represent collinear pairs in *AhALDH*s, and a gray background represents all collinear blocks. **(B)** Collinear analysis of *AhALDH*s with *Arabidopsis* members. The red circles represent groundnut chromosomes, while green circles represent *Arabidopsis* chromosomes, and the green lines represent collinear gene pairs.

Fifty-two pairs of collinear *AhALDH*s were identified. Most of the *AhALDH* members had more than two collinear pairs; *AhALDH2B3* had five collinear pairs, which revealed that *AhALDH2B3* might have a longer and more pivotal evolutionary relationship (**Figure 6A**). Compared with the *Arabidopsis* members, *AhALDH*s had 24 collinear pairs and likely similar functions to those of *Arabidopsis* collinear genes. Interestingly, both collinear *Arabidopsis* members and *AhALDH* members were not distributed on the last chromosome, which potentially reveals some characteristics of the ALDH members (**Figures 2**, **6B**).

### 3.7 GO and KEGG enrichment analysis of AhALDHs

**Figure 7:**
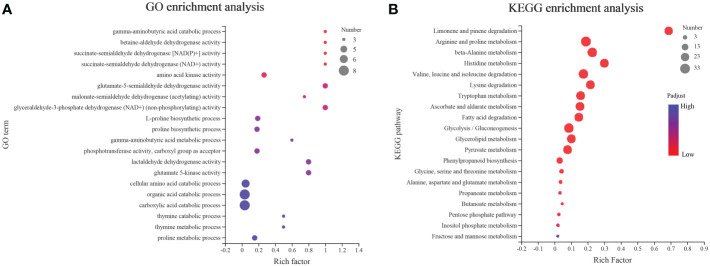
GO and KEGG enrichment analysis of *AhALDH* members. The size of each circle represents the number of *AhALDH*s. The transition in color from blue to red represents the P-value from high to low. **(A)** GO enrichment analysis of *AhALDH*s. **(B)** KEGG enrichment analysis of *AhALDH*s.

The GO and KEGG enrichment analyses (**Figure 7**) were conducted to understand the function of *AhALDH*s. The *AhALDH*s were significantly (*P* < 0.05) enriched in 81 GO terms (**Table S4**); the top-10 GO terms were enriched in semialdehyde dehydrogenase activity (6) and amino acid-related terms (4), suggesting that *AhALDH*s might participate in the growth of plants (**Figure 7A**). The analysis further revealed that the *AhALDH*s were significantly (*P* < 0.05) enriched in 20 pathways (**Table S5**); eight pathways were related to amino acid metabolism, indicating the role of *AhALDH*s in the biosynthesis of amino acids (**Figure 7B**). These results indicated that *AhALDH*s might exercise function through amino acid pathways.

### 3.8 Tissue-specific expression pattern analysis of *AhALDHs*

**Figure 8:**
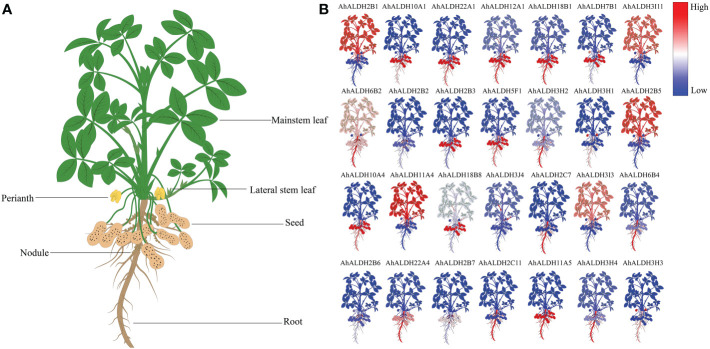
Expression pattern analysis of *AhALDH*s in different tissues. **(A)** Schematic diagram of different groundnut tissues; plants were divided into root, nodule, seed, perianth, and leaf. **(B)** Expression levels of *AhALDH*s. A change in color from blue to red represents *ALDH* levels from low to high.

The expression pattern of 28 *AhALDH* members from different tissues encompassing all subfamilies was extracted from the Phytozome database, and a heatmap was drawn using TBtools. The results showed that the expression of *AhALDHs* was tissue-specific (**Figure 8**). Some *AhALDHs* had a higher expression level (such as *AhALDH12A1*, *AhALDH18B1*, *AhALDH6B2*, *AhALDH2C7*, *AhALDH6B4*, *AhALDH2C11*, and *AhALDH11A5*) in roots than in other tissues; therefore, roots should be used as a target tissue of *AhALDHs* for further study.

### 3.9 ALDH activity under saline-alkali stress

**Figure 9:**
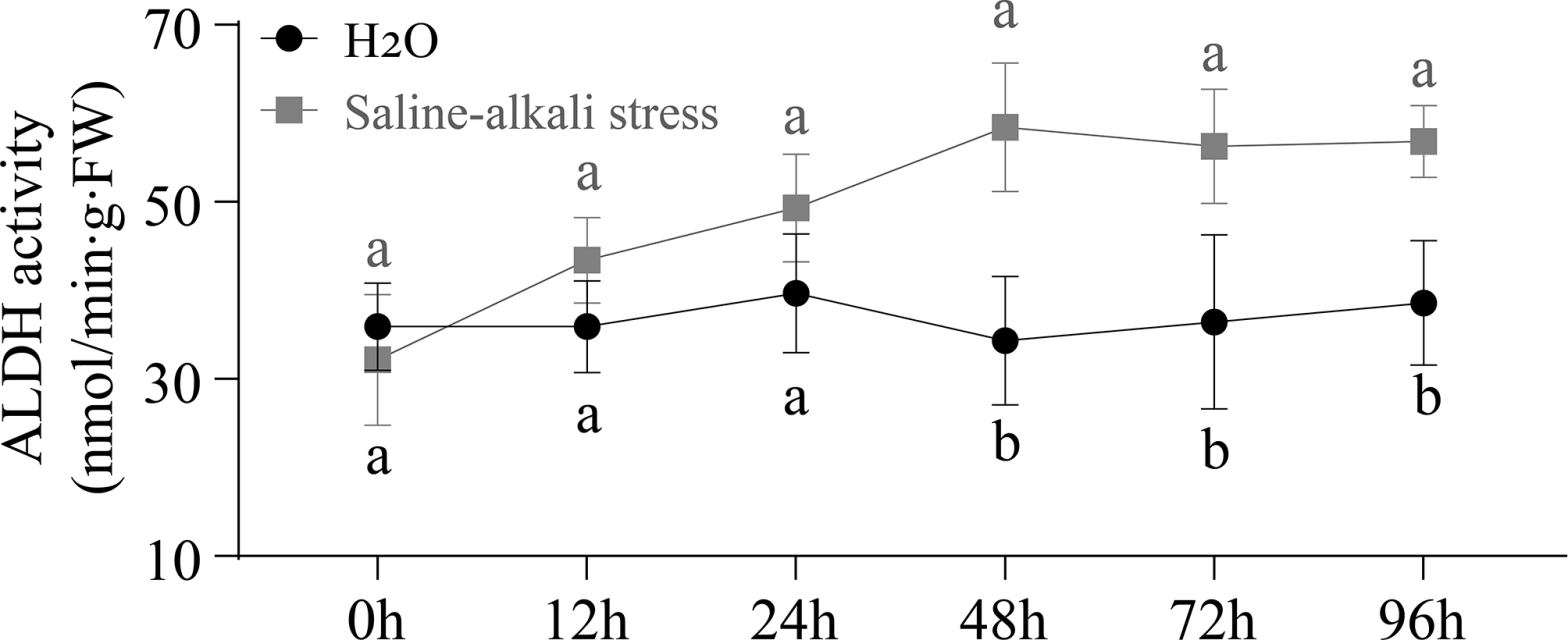
Variation curve of ALDH enzyme activity under control and saline-alkali stress treatment. Black line represents ALDH enzyme activity in H_2_O treatment at the sprout stage, while gray line represents ALDH enzyme activity in saline-alkali stress treatment.

The ALDH activity was determined under control and stress conditions at 0, 12, 24, 48, 72, and 96 h (**Figure 9**). The ALDH activity showed no obvious trend under the control conditions, but the data of activity under saline-alkali stress had increased. The results showed that the ALDH activity began to significantly change at 48 h (*P* < 0.05). All these results illustrated that 48 h could be used as an simulated stress time for testing the expression of *AhALDH* members according to ALDH Activity.

### 3.10 Expression analysis under saline-alkali stress

**Figure 10:**
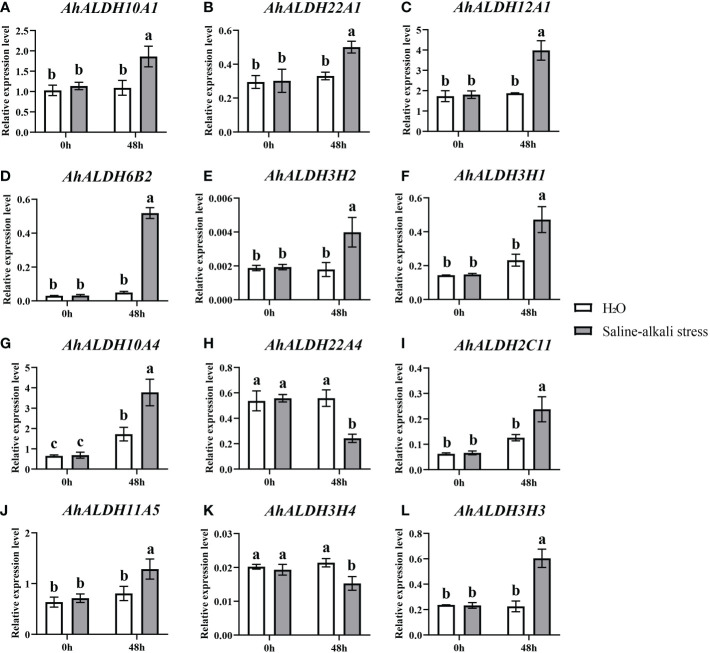
Expression pattern analysis of 12 *AhALDHs* under saline-alkali stress at 0h and 48h. White columns show the expression in the H_2_O at the sprout stage, while gray columns show the expression in the saline-alkali stress treatment. **(A–L)** The expression analysis of 12 AhALDH members.

Twelve *AhALDH*s with higher expression in the roots at the sprout stage, based on the tissue-specific expression pattern analysis, were selected for qRT-PCR analysis under stress, and 48 h was used as the sampling time. The expression of these members significantly differed between the control and stress treatment (*P* < 0.05). Some *AhALDH* members, *AhALDH10A1, AhALDH22A1, AhALDH12A1, AhALDH6B2, AhALDH3H2, AhALDH3H1, AhALDH10A4, AhALDH2C11, AhALDH11A5*, and *AhALDH3H3*, were upregulated under saline-alkali stress, while others, *AhALDH22A4* and *AhALDH3H4*, were downregulated. These results suggested the role of these members in plant response to abiotic stress; some might be involved in positive regulation, while some might be involved in negative regulation (**Figures 10A-L**).

## 4 Discussion

Members of the ALDH superfamily have previously been identified in several species (Brocker et al., 2013): 9 ALDH members were identified in *Arabidopsis* (Kirch et al., 2004), 20 were reported for rice (Gao and Han, 2009), 23 for grape (Zhou et al., 2012), and 19 for tomato (*Solanum lycopersicum*) (Zhang et al., 2012); 28 for maize (Zhou et al., 2012), 19 for sorghum (*Sorghum bicolor*) (Islam et al., 2022), and 53 for soybean (Wang et al., 2017). In the present study, 71 ALDH members were identified through the reference genome (*Arachis hypogaea* v1.0). Such a large number of ALDH members might be related to the large size of the genome and the evolution of the genes (Wang et al., 2017). The evolutionary relationship analysis resolved these members into 10 subfamilies (Man et al., 2020); similarly, *VvALDHs*, *ZmALDHs*, and *GhALDHs* were divided into 10 subfamilies (Zhang et al., 2012; Zhou et al., 2012; Dong et al., 2017), which reveals that 10 subfamilies express the evolutionary relationship characteristics of ALDH members. The evolutionary relationship of seven species confirmed the accuracy of the member classification into 10 subfamilies.

Motif analysis can be used to reveal the function of members according to protein sequences, in which the similar kinds of motifs may have a similar function (Bailey et al., 2015; Liu et al., 2021). The motif analysis of AhALDHs showed that the members in each subfamily shared similar kinds of motifs; these results are in accordance with those reported for ALDH members in soybean (Wang et al., 2017). In *cis*-acting element analysis, *AhALDH*s with TGA-element, ABRE, P-box, GARE-motif, TCA-element, AT-rich sequence, SARE, TATC-box, and AuxRR-core were related to hormones, whereas LTR, MBS, ARE, and GC-motif elements were associated with abiotic stress response, suggesting that *AhALDH*s are related to abiotic stress and hormones. Similarly, the elements LTR, MBS, ABRE, P-box, TCA-element, and AuxRR-core were found in *SbALDHs* (Islam et al., 2022), whereas LTR, MBS, ARE, ARBE, and TCA-element were predicted in *GhALDH*s (Guo et al., 2017; Yang et al., 2019). Thus, *cis*-acting elements such as LTR, MBS, ARE, ARBE, and TCA-element are common *cis*-acting elements among ALDH members, contributing to the role of *AhALDH*s in plant response to abiotic stress and hormone interactions (Chen Z, et al., 2014). Some *AhALDH*s were colinear with *Arabidopsis* genes; for example, *AhALDH10A2* was colinear with *AT1G74920*, which is involved in plant response to salt and drought (Missihoun et al., 2011); *AhALDH18B9* was colinear with *AT2G39800* (*P5CS1*), which acts in abiotic stress response (salt, oxidative, ABA, desiccation, and water deprivation) and participates in proline biosynthesis (Yoshiba et al., 1995; Yoshiba et al., 1999; Huang et al., 2008; Székely et al., 2008); and *AhALDH2B2* and *AhALDH2B6* were colinear with *AT1G23800* (*ALDH2B7*), a gene that responds to drought and abscisic acid signal (Depuydt and Vandepoele, 2021). These relationships indicated the role *AhALDH*s play in plant response to abiotic stresses. *AhALDH*s were enriched in semialdehyde dehydrogenase activity (GO results) and amino acid metabolism (GO and KEGG analysis). Semialdehyde dehydrogenase activity is closely related with abiotic stress and increases stress conditions (Wei et al., 2021; Zhang et al., 2021). Amino acid metabolism usually intensifies during the growth and development of plants especially under stress, such as GST family members (Batista-Silva et al., 2019; Yang et al., 2019). Some amino acids, such as proline, have been used as a parameter for assessing damage to plants under abiotic stress (Ghosh et al., 2022). The enrichment analysis further corroborated the correlation between *AhALDHs* and abiotic stress.

The expression of several ALDH members, such as *GhALDH*s, *GmALDH*s, and *SiALDH*s, shows a tissue-specific pattern and is higher in roots (Dong et al., 2017; Wang et al., 2017). A similar pattern was observed in the present study, suggesting that the root system is a suitable target for studying ALDH members. Some ALDH members, such as *BrALDH7B2*, were predicted to function in stress response (Gautam et al., 2019). Similar to that of *VvALDHs*, *SiALDHs*, and *CaALDHs* (Zhang et al., 2012; Chen C, et al., 2014; Carmona-Molero et al., 2021), the expression of some *AhALDHs* (such as *AhALDH10A1*, *AhALDH22A1*, *AhALDH12A1*, or *AhALDH6B2*) was significantly upregulated, while it was downregulated in others (such as *AhALDH22A4* and *AhALDH3H4*) under alkali-stress conditions, which these upregulated six members could be considered as cluster genes involved in resistance to abiotic stress.

## 5 Conclusion

In this study, 71 ALDH members were identified from the groundnut reference genome and classified into 10 subfamilies, with similar motifs and gene structure. *AhALDHs* were associated with abiotic stress response and hormones *via cis*-acting elements. The results of collinearity and enrichment analysis (including GO and KEGG pathway analysis) revealed that *AhALDHs* are involved in plant response to stress and their expression is tissue-specific. The root system is a target tissue suitable for studying *AhALDHs*, and upregulated members can be used as candidate *AhALDHs* members (such *AhALDH10A1*, *AhALDH22A1*, *AhALDH12A1*, or *AhALDH6B2*) involved in resistance to abiotic stress in future research. This study provides insights into *AhALDHs* and the basis for further research of groundnut.

## Data availability statement

The datasets presented in this study can be found in online repositories. The names of the repository/repositories and accession number(s) can be found in the article/**Supplementary Material**.

## Author contributions

Conceptualization: XZ and JZ; methodology: LCa and GY; software: CR; validation: YG, JR, HR, and SZ; formal analysis and investigation: LCh and YZ; writing—review and editing: LW, XZ and YZ; funding acquisition: YZ. All authors contributed to the article and approved the submitted version.

## Supplementary material

Supplementary Table 1 The primers sequence of ALDH members in groundnut.

Supplementary Table 2 The detailed information of ALDH members in groundnut.

Supplementary Table 3 The information of *cis*-acting elements.

Supplementary Table 4 The Gene Ontology (GO) terms enriched in *AhALDHs*.

Supplementary Table 5 The Kyoto Encyclopedia of Genes and Genomes (KEGG) pathways enriched in *AhALDHs.*
