# Supplementary material for: Genome-wide characterization of aldehyde dehydrogenase gene family members in groundnut (Arachis hypogaea) and the analysis under saline-alkali stress
Source: Front Plant Sci. 2023 Feb 16;14:1097001. doi: 10.3389/fpls.2023.1097001 (PMC9978533; doi:10.3389/fpls.2023.1097001)
Supplement: Supplementary Table 1 — The primers sequence of ALDH members in groundnut. [file DataSheet_1.zip › table/Table 2.DOCX]

Table S2

| No. | Gene name | Phytozome ID | Chr | Start Position  (bp) | End Position  (bp) | CDS  (bp) | ORF (aa) | Molecular Weight  (kD) | Isoelectric Point  (PI) |
| --- | --- | --- | --- | --- | --- | --- | --- | --- | --- |
| 1 | AhALDH3F1 | arahy.Tifrunner.gnm1.ann1.6N07W3.1 | Chr01 | 1621995 | 1635244 | 1272 | 423 | 47.06 | 8.62 |
| 2 | AhALDH2B1 | arahy.Tifrunner.gnm1.ann1.Q3LZKR.1 | Chr01 | 86917619 | 86923061 | 1590 | 529 | 58.14 | 8.24 |
| 3 | AhALDH10A1 | arahy.Tifrunner.gnm1.ann1.M4G3QU.1 | Chr01 | 92799436 | 92802981 | 1512 | 503 | 54.55 | 5.34 |
| 4 | AhALDH11A1 | arahy.Tifrunner.gnm1.ann1.U9DM18.1 | Chr01 | 97729994 | 97733818 | 1491 | 496 | 53.08 | 7.08 |
| 5 | AhALDH22A1 | arahy.Tifrunner.gnm1.ann1.F4QS6F.1 | Chr01 | 100841983 | 100847425 | 1794 | 597 | 66.12 | 7.17 |
| 6 | AhALDH12A1 | arahy.Tifrunner.gnm1.ann1.0II3I9.1 | Chr01 | 111707918 | 111714684 | 1632 | 543 | 59.98 | 6.97 |
| 7 | AhALDH18B1 | arahy.Tifrunner.gnm1.ann1.AXAR8E.1 | Chr02 | 84221825 | 84236912 | 2160 | 719 | 78.46 | 5.96 |
| 8 | AhALDH3J1 | arahy.Tifrunner.gnm1.ann1.7DG5CM.1 | Chr03 | 3378209 | 3381536 | 1551 | 516 | 57.84 | 9.03 |
| 9 | AhALDH2C1 | arahy.Tifrunner.gnm1.ann1.D91PU0.1 | Chr03 | 35199335 | 35204490 | 1377 | 458 | 49.75 | 6.05 |
| 10 | AhALDH7B1 | arahy.Tifrunner.gnm1.ann1.159H25.1 | Chr03 | 49134047 | 49139390 | 1485 | 494 | 52.97 | 5.36 |
| 11 | AhALDH3I1 | arahy.Tifrunner.gnm1.ann1.3M3TNE.2 | Chr03 | 119187882 | 119191170 | 1626 | 541 | 59.65 | 6.50 |
| 12 | AhALDH6B1 | arahy.Tifrunner.gnm1.ann1.WZ366M.1 | Chr03 | 125424722 | 125430319 | 1620 | 539 | 57.77 | 8.32 |
| 13 | AhALDH6B2 | arahy.Tifrunner.gnm1.ann1.QCB2A1.1 | Chr03 | 131985657 | 131992421 | 2376 | 791 | 87.04 | 5.38 |
| 14 | AhALDH2B2 | arahy.Tifrunner.gnm1.ann1.ZMIE54.1 | Chr03 | 133228798 | 133232667 | 1710 | 569 | 62.30 | 6.01 |
| 15 | AhALDH22A2 | arahy.Tifrunner.gnm1.ann1.EEWD1J.1 | Chr04 | 13878597 | 13885101 | 1791 | 596 | 65.54 | 7.21 |
| 16 | AhALDH18B2 | arahy.Tifrunner.gnm1.ann1.1W477Y.1 | Chr04 | 83121290 | 83131526 | 2103 | 700 | 75.72 | 6.35 |
| 17 | AhALDH11A2 | arahy.Tifrunner.gnm1.ann1.NABN1H.1 | Chr04 | 97303623 | 97312806 | 1119 | 373 | 42.17 | 9.10 |
| 18 | AhALDH2C2 | arahy.Tifrunner.gnm1.ann1.08HL65.1 | Chr05 | 98029222 | 98034476 | 1521 | 506 | 55.30 | 5.69 |
| 19 | AhALDH2C3 | arahy.Tifrunner.gnm1.ann1.ZE0PKC.1 | Chr05 | 98123714 | 98134130 | 1548 | 515 | 54.64 | 6.16 |
| 20 | AhALDH2C4 | arahy.Tifrunner.gnm1.ann1.3LD4UL.1 | Chr05 | 98144457 | 98156713 | 1437 | 478 | 52.23 | 6.48 |
| 21 | AhALDH2B3 | arahy.Tifrunner.gnm1.ann1.8CP0UI.1 | Chr05 | 106050833 | 106054320 | 1629 | 542 | 58.76 | 6.73 |
| 22 | AhALDH10A2 | arahy.Tifrunner.gnm1.ann1.QCS9HL.1 | Chr05 | 107670814 | 107678580 | 1563 | 520 | 56.43 | 5.73 |
| 23 | AhALDH10A3 | arahy.Tifrunner.gnm1.ann1.KIIE1S.1 | Chr06 | 48275513 | 48278993 | 1665 | 555 | 62.59 | 7.64 |
| 24 | AhALDH3J2 | arahy.Tifrunner.gnm1.ann1.TUX9ZC.1 | Chr06 | 101553110 | 101555707 | 957 | 319 | 36.14 | 5.70 |
| 25 | AhALDH11A3 | arahy.Tifrunner.gnm1.ann1.UG6CYA.1 | Chr06 | 109851755 | 109856260 | 1434 | 477 | 51.24 | 7.88 |
| 26 | AhALDH5F1 | arahy.Tifrunner.gnm1.ann1.D1NSDN.1 | Chr07 | 40337127 | 40348904 | 1599 | 532 | 57.09 | 8.66 |
| 27 | AhALDH3H2 | arahy.Tifrunner.gnm1.ann1.0B7PBW.1 | Chr08 | 10683713 | 10689736 | 1479 | 492 | 53.58 | 8.89 |
| 28 | AhALDH3H1 | arahy.Tifrunner.gnm1.ann1.Z6ZXKG.1 | Chr08 | 10683713 | 10689736 | 1473 | 490 | 53.60 | 8.58 |
| 29 | AhALDH3J3 | arahy.Tifrunner.gnm1.ann1.EGRI7M.1 | Chr08 | 26738054 | 26743938 | 1503 | 500 | 55.46 | 9.22 |
| 30 | AhALDH3I2 | arahy.Tifrunner.gnm1.ann1.05HDV3.1 | Chr08 | 48039556 | 48047021 | 1320 | 439 | 48.37 | 8.32 |
| 31 | AhALDH2B4 | arahy.Tifrunner.gnm1.ann1.3JA9V5.2 | Chr09 | 19511062 | 19516959 | 2208 | 736 | 83.00 | 8.86 |
| 32 | AhALDH3F2 | arahy.Tifrunner.gnm1.ann1.6ZS5B3.1 | Chr11 | 13465653 | 13490064 | 1149 | 382 | 42.37 | 8.81 |
| 33 | AhALDH2C5 | arahy.Tifrunner.gnm1.ann1.54H2MY.1 | Chr11 | 73567893 | 73628484 | 909 | 303 | 32.48 | 5.82 |
| 34 | AhALDH2B5 | arahy.Tifrunner.gnm1.ann1.W2MY31.1 | Chr11 | 111918811 | 111923767 | 1623 | 540 | 59.40 | 8.41 |
| 35 | AhALDH10A4 | arahy.Tifrunner.gnm1.ann1.JTTA2P.1 | Chr11 | 115179184 | 115182727 | 1512 | 503 | 54.58 | 5.34 |
| 36 | AhALDH12A2 | arahy.Tifrunner.gnm1.ann1.77YH77.1 | Chr11 | 121442283 | 121449041 | 1632 | 556 | 61.77 | 6.26 |
| 37 | AhALDH22A3 | arahy.Tifrunner.gnm1.ann1.6QU7U5.1 | Chr11 | 142659569 | 142665041 | 1794 | 597 | 66.10 | 7.17 |
| 38 | AhALDH11A4 | arahy.Tifrunner.gnm1.ann1.5CRU8P.1 | Chr11 | 146562753 | 146566371 | 1320 | 439 | 47.07 | 8.20 |
| 39 | AhALDH18B8 | arahy.Tifrunner.gnm1.ann1.WS4P7I.1 | Chr12 | 97927141 | 97941815 | 2259 | 752 | 81.99 | 6.07 |
| 40 | AhALDH3J4 | arahy.Tifrunner.gnm1.ann1.8D8LNX.2 | Chr13 | 5634789 | 5637696 | 1437 | 478 | 53.44 | 9.00 |
| 41 | AhALDH2C6 | arahy.Tifrunner.gnm1.ann1.AWX4M7.1 | Chr13 | 37593111 | 37595806 | 969 | 323 | 35.05 | 6.09 |
| 42 | AhALDH2C7 | arahy.Tifrunner.gnm1.ann1.XE3XSF.1 | Chr13 | 37603569 | 37617894 | 1347 | 448 | 48.47 | 5.51 |
| 43 | AhALDH7B2 | arahy.Tifrunner.gnm1.ann1.XD58IC.1 | Chr13 | 51529478 | 51534838 | 1485 | 494 | 53.02 | 5.36 |
| 44 | AhALDH3I3 | arahy.Tifrunner.gnm1.ann1.BZ0RB3.3 | Chr13 | 121830706 | 121836541 | 1554 | 517 | 57.23 | 7.08 |
| 45 | AhALDH6B3 | arahy.Tifrunner.gnm1.ann1.HY8A1I.1 | Chr13 | 129317576 | 129323260 | 1620 | 539 | 57.84 | 8.66 |
| 46 | AhALDH18B9 | arahy.Tifrunner.gnm1.ann1.NA5QFN.1 | Chr13 | 131949299 | 131955109 | 2130 | 709 | 77.13 | 6.20 |
| 47 | AhALDH6B4 | arahy.Tifrunner.gnm1.ann1.0HR61J.5 | Chr13 | 134507033 | 134515588 | 2007 | 668 | 73.91 | 5.71 |
| 48 | AhALDH2B6 | arahy.Tifrunner.gnm1.ann1.3CBS5R.1 | Chr13 | 135828679 | 135832617 | 1710 | 569 | 62.28 | 5.96 |
| 49 | AhALDH22A4 | arahy.Tifrunner.gnm1.ann1.CLI46N.1 | Chr14 | 15360479 | 15367089 | 1785 | 594 | 65.26 | 6.55 |
| 50 | AhALDH18B10 | arahy.Tifrunner.gnm1.ann1.V8868B.1 | Chr14 | 94572345 | 94584668 | 2100 | 699 | 75.71 | 6.94 |
| 51 | AhALDH10A5 | arahy.Tifrunner.gnm1.ann1.VJ6UAM.1 | Chr14 | 102564973 | 102595570 | 1365 | 455 | 50.67 | 6.84 |
| 52 | AhALDH3J5 | arahy.Tifrunner.gnm1.ann1.UE0WIX.1 | Chr15 | 9783282 | 9786494 | 1239 | 413 | 46.45 | 5.28 |
| 53 | AhALDH3J6 | arahy.Tifrunner.gnm1.ann1.XYS3VK.2 | Chr15 | 102519473 | 102522712 | 1272 | 424 | 47.61 | 5.23 |
| 54 | AhALDH2C8 | arahy.Tifrunner.gnm1.ann1.EX0EXI.1 | Chr15 | 107032262 | 107036666 | 1299 | 433 | 48.15 | 5.21 |
| 55 | AhALDH10A6 | arahy.Tifrunner.gnm1.ann1.59SDUF.1 | Chr15 | 129771851 | 129783735 | 1512 | 503 | 54.47 | 5.25 |
| 56 | AhALDH2B7 | arahy.Tifrunner.gnm1.ann1.STWC1J.1 | Chr15 | 133711196 | 133714631 | 1629 | 542 | 58.70 | 7.10 |
| 57 | AhALDH2C9 | arahy.Tifrunner.gnm1.ann1.QE2QJ0.1 | Chr15 | 147414338 | 147423347 | 1488 | 495 | 53.81 | 5.99 |
| 58 | AhALDH2C10 | arahy.Tifrunner.gnm1.ann1.SY7QJV.1 | Chr15 | 147614320 | 147624227 | 1515 | 504 | 54.64 | 6.16 |
| 59 | AhALDH2C11 | arahy.Tifrunner.gnm1.ann1.J17R0W.1 | Chr15 | 147754118 | 147758911 | 1521 | 506 | 55.25 | 5.66 |
| 60 | AhALDH2C12 | arahy.Tifrunner.gnm1.ann1.U0A30G.1 | Chr15 | 149234021 | 149237667 | 1116 | 372 | 40.95 | 4.91 |
| 61 | AhALDH3J7 | arahy.Tifrunner.gnm1.ann1.WJV1YC.1 | Chr16 | 101783070 | 101785825 | 915 | 305 | 34.38 | 6.67 |
| 62 | AhALDH11A5 | arahy.Tifrunner.gnm1.ann1.U399DB.1 | Chr16 | 148336260 | 148340560 | 1542 | 513 | 55.66 | 8.08 |
| 63 | AhALDH10A7 | arahy.Tifrunner.gnm1.ann1.E23JR7.1 | Chr17 | 87822491 | 87826164 | 954 | 318 | 36.34 | 9.61 |
| 64 | AhALDH3H4 | arahy.Tifrunner.gnm1.ann1.JXX0IG.1 | Chr17 | 94744757 | 94751700 | 1665 | 554 | 60.32 | 8.91 |
| 65 | AhALDH3H3 | arahy.Tifrunner.gnm1.ann1.9H5ER3.1 | Chr17 | 126985177 | 126990463 | 1473 | 490 | 53.49 | 8.39 |
| 66 | AhALDH3J8 | arahy.Tifrunner.gnm1.ann1.A31U7C.1 | Chr18 | 2993601 | 2998749 | 1329 | 442 | 49.26 | 9.07 |
| 67 | AhALDH10A8 | arahy.Tifrunner.gnm1.ann1.1C51KT.1 | Chr18 | 47510508 | 47515626 | 1368 | 456 | 50.83 | 6.33 |
| 68 | AhALDH5F2 | arahy.Tifrunner.gnm1.ann1.15VWWC.1 | Chr18 | 99430471 | 99442360 | 1620 | 539 | 57.83 | 8.46 |
| 69 | AhALDH3I4 | arahy.Tifrunner.gnm1.ann1.P5XJ25.1 | Chr18 | 132291124 | 132296510 | 1281 | 426 | 46.93 | 8.05 |
| 70 | AhALDH2B8 | arahy.Tifrunner.gnm1.ann1.HW3SC4.1 | Chr19 | 25062258 | 25068768 | 2352 | 784 | 88.83 | 8.85 |
| 71 | AhALDH3J9 | arahy.Tifrunner.gnm1.ann1.DF36MW.1 | Chr19 | 144593551 | 144596803 | 1200 | 400 | 45.04 | 4.95 |
