# Supplementary material for: Genome-wide characterization of aldehyde dehydrogenase gene family members in groundnut (Arachis hypogaea) and the analysis under saline-alkali stress
Source: Front Plant Sci. 2023 Feb 16;14:1097001. doi: 10.3389/fpls.2023.1097001 (PMC9978533; doi:10.3389/fpls.2023.1097001)
Supplement: Supplementary Table 1 — The primers sequence of ALDH members in groundnut. [file DataSheet_1.zip › table/Table 3.DOCX]

Table S3

| Element name | Annotation information |
| --- | --- |
| TGA-element | auxin-responsive element |
| ABRE | cis-acting element involved in the abscisic acid responsiveness |
| LTR | cis-acting element involved in low-temperature responsiveness |
| CAT-box | cis-acting regulatory element related to meristem expression |
| MBS | MYB binding site involved in drought-inducibility |
| P-box | gibberellin-responsive element |
| ARE | cis-acting regulatory element essential for the anaerobic induction |
| GARE-motif | gibberellin-responsive element |
| TCA-element | cis-acting element involved in salicylic acid responsiveness |
| AT-rich sequence | element for maximal elicitor-mediated activation (2copies) |
| SARE | cis-acting element involved in salicylic acid responsiveness |
| TATC-box | cis-acting element involved in gibberellin-responsiveness |
| AuxRR-core | cis-acting regulatory element involved in auxin responsiveness |
| GC-motif | enhancer-like element involved in anoxic specific inducibility |
| MBSI | MYB binding site involved in flavonoid biosynthetic genes regulation |
