# Supplementary material for: Genome-wide characterization of aldehyde dehydrogenase gene family members in groundnut (Arachis hypogaea) and the analysis under saline-alkali stress
Source: Front Plant Sci. 2023 Feb 16;14:1097001. doi: 10.3389/fpls.2023.1097001 (PMC9978533; doi:10.3389/fpls.2023.1097001)
Supplement: Supplementary Table 1 — The primers sequence of ALDH members in groundnut. [file DataSheet_1.zip › table/Table 4.DOCX]

Table S4

| No. | GO ID | Term Type | Description | Pvalue | Padjust |
| --- | --- | --- | --- | --- | --- |
| 3 | GO:0009450 | BP | gamma-aminobutyric acid catabolic process | 2.89E-09 | 5.60E-06 |
| 3 | GO:0008802 | MF | betaine-aldehyde dehydrogenase activity | 2.89E-09 | 5.60E-06 |
| 3 | GO:0009013 | MF | succinate-semialdehyde dehydrogenase [NAD(P)+] activity | 2.89E-09 | 5.60E-06 |
| 3 | GO:0004777 | MF | succinate-semialdehyde dehydrogenase (NAD+) activity | 2.89E-09 | 5.60E-06 |
| 4 | GO:0019202 | MF | amino acid kinase activity | 5.42E-09 | 8.39E-06 |
| 4 | GO:0004350 | MF | glutamate-5-semialdehyde dehydrogenase activity | 1.16E-08 | 1.12E-05 |
| 3 | GO:0018478 | MF | malonate-semialdehyde dehydrogenase (acetylating) activity | 1.16E-08 | 1.12E-05 |
| 4 | GO:0043878 | MF | glyceraldehyde-3-phosphate dehydrogenase (NAD+) (non-phosphorylating) activity | 1.16E-08 | 1.12E-05 |
| 4 | GO:0055129 | BP | L-proline biosynthetic process | 2.36E-08 | 1.60E-05 |
| 4 | GO:0006561 | BP | proline biosynthetic process | 2.88E-08 | 1.60E-05 |
| 3 | GO:0009448 | BP | gamma-aminobutyric acid metabolic process | 2.89E-08 | 1.60E-05 |
| 4 | GO:0016774 | MF | phosphotransferase activity, carboxyl group as acceptor | 2.88E-08 | 1.60E-05 |
| 4 | GO:0008911 | MF | lactaldehyde dehydrogenase activity | 2.89E-08 | 1.60E-05 |
| 4 | GO:0004349 | MF | glutamate 5-kinase activity | 2.89E-08 | 1.60E-05 |
| 6 | GO:0009063 | BP | cellular amino acid catabolic process | 4.41E-08 | 2.16E-05 |
| 8 | GO:0016054 | BP | organic acid catabolic process | 5.02E-08 | 2.16E-05 |
| 8 | GO:0046395 | BP | carboxylic acid catabolic process | 5.02E-08 | 2.16E-05 |
| 3 | GO:0006210 | BP | thymine catabolic process | 5.77E-08 | 2.16E-05 |
| 3 | GO:0019859 | BP | thymine metabolic process | 5.77E-08 | 2.16E-05 |
| 4 | GO:0006560 | BP | proline metabolic process | 5.86E-08 | 2.16E-05 |
| 4 | GO:0033721 | MF | aldehyde dehydrogenase (NADP+) activity | 5.77E-08 | 2.16E-05 |
| 67 | GO:0016903 | MF | oxidoreductase activity, acting on the aldehyde or oxo group of donors | 7.08E-08 | 2.49E-05 |
| 5 | GO:0009414 | BP | response to water deprivation | 8.78E-08 | 2.96E-05 |
| 3 | GO:0006574 | BP | valine catabolic process | 1.01E-07 | 3.26E-05 |
| 5 | GO:0009415 | BP | response to water | 1.14E-07 | 3.54E-05 |
| 28 | GO:0004029 | MF | aldehyde dehydrogenase (NAD+) activity | 1.23E-07 | 3.66E-05 |
| 8 | GO:0044282 | BP | small molecule catabolic process | 1.33E-07 | 3.82E-05 |
| 5 | GO:0001101 | BP | response to acid chemical | 1.46E-07 | 4.03E-05 |
| 3 | GO:0006208 | BP | pyrimidine nucleobase catabolic process | 1.61E-07 | 4.03E-05 |
| 4 | GO:0004491 | MF | methylmalonate-semialdehyde dehydrogenase (acylating) activity | 1.59E-07 | 4.03E-05 |
| 30 | GO:0004030 | MF | aldehyde dehydrogenase [NAD(P)+] activity | 1.59E-07 | 4.03E-05 |
| 4 | GO:0008250 | CC | oligosaccharyltransferase complex | 1.80E-07 | 4.36E-05 |
| 9 | GO:0004028 | MF | 3-chloroallyl aldehyde dehydrogenase activity | 2.41E-07 | 5.67E-05 |
| 78 | GO:0016491 | MF | oxidoreductase activity | 4.01E-07 | 9.14E-05 |
| 67 | GO:0016620 | MF | oxidoreductase activity, acting on the aldehyde or oxo group of donors, NAD or NADP as acceptor | 5.21E-07 | 0.000115271 |
| 3 | GO:0046113 | BP | nucleobase catabolic process | 6.31E-07 | 0.000135701 |
| 10 | GO:0006520 | BP | cellular amino acid metabolic process | 6.50E-07 | 0.000136035 |
| 15 | GO:0006081 | BP | cellular aldehyde metabolic process | 8.48E-07 | 0.000172888 |
| 78 | GO:0003824 | MF | catalytic activity | 1.26E-06 | 0.000249955 |
| 8 | GO:1901605 | BP | alpha-amino acid metabolic process | 1.62E-06 | 0.000305605 |
| 3 | GO:0043649 | BP | dicarboxylic acid catabolic process | 1.94E-06 | 0.000318681 |
| 2 | GO:0042560 | BP | pteridine-containing compound catabolic process | 2.06E-06 | 0.000318681 |
| 2 | GO:0009397 | BP | folic acid-containing compound catabolic process | 2.06E-06 | 0.000318681 |
| 2 | GO:0009258 | BP | 10-formyltetrahydrofolate catabolic process | 2.06E-06 | 0.000318681 |
| 78 | GO:0003674 | MF | molecular_function | 1.87E-06 | 0.000318681 |
| 2 | GO:0001758 | MF | retinal dehydrogenase activity | 2.06E-06 | 0.000318681 |
| 2 | GO:0004043 | MF | L-aminoadipate-semialdehyde dehydrogenase activity | 2.06E-06 | 0.000318681 |
| 2 | GO:0050269 | MF | coniferyl-aldehyde dehydrogenase activity | 2.06E-06 | 0.000318681 |
| 2 | GO:0016155 | MF | formyltetrahydrofolate dehydrogenase activity | 2.06E-06 | 0.000318681 |
| 3 | GO:0006573 | BP | valine metabolic process | 2.32E-06 | 0.000333462 |
| 3 | GO:0009083 | BP | branched-chain amino acid catabolic process | 4.37E-06 | 0.000604331 |
| 4 | GO:0006487 | BP | protein N-linked glycosylation | 4.76E-06 | 0.000642737 |
| 5 | GO:0009064 | BP | glutamine family amino acid metabolic process | 4.81E-06 | 0.000642737 |
| 4 | GO:0009084 | BP | glutamine family amino acid biosynthetic process | 5.85E-06 | 0.000767637 |
| 12 | GO:0019752 | BP | carboxylic acid metabolic process | 9.04E-06 | 0.001167887 |
| 12 | GO:0043436 | BP | oxoacid metabolic process | 1.11E-05 | 0.001410865 |
| 3 | GO:0072529 | BP | pyrimidine-containing compound catabolic process | 1.14E-05 | 0.001427122 |
| 12 | GO:0006082 | BP | organic acid metabolic process | 1.23E-05 | 0.001491015 |
| 2 | GO:0009256 | BP | 10-formyltetrahydrofolate metabolic process | 1.23E-05 | 0.001491015 |
| 8 | GO:1901565 | BP | organonitrogen compound catabolic process | 1.85E-05 | 0.002210211 |
| 4 | GO:0097305 | BP | response to alcohol | 2.38E-05 | 0.002707523 |
| 4 | GO:0009737 | BP | response to abscisic acid | 2.38E-05 | 0.002707523 |
| 4 | GO:1901606 | BP | alpha-amino acid catabolic process | 2.54E-05 | 0.002856916 |
| 5 | GO:0010035 | BP | response to inorganic substance | 3.86E-05 | 0.004277066 |
| 3 | GO:0006206 | BP | pyrimidine nucleobase metabolic process | 6.80E-05 | 0.007322834 |
| 5 | GO:0003779 | MF | actin binding | 7.98E-05 | 0.008356288 |
| 4 | GO:0033993 | BP | response to lipid | 8.48E-05 | 0.008762674 |
| 5 | GO:0046700 | BP | heterocycle catabolic process | 9.16E-05 | 0.009337632 |
| 5 | GO:0044270 | BP | cellular nitrogen compound catabolic process | 0.000100162 | 0.01007859 |
| 3 | GO:0009081 | BP | branched-chain amino acid metabolic process | 0.000181257 | 0.017776952 |
| 2 | GO:0042219 | BP | cellular modified amino acid catabolic process | 0.00021332 | 0.020156124 |
| 3 | GO:0016459 | CC | myosin complex | 0.000211392 | 0.020156124 |
| 5 | GO:1901361 | BP | organic cyclic compound catabolic process | 0.000274239 | 0.025167492 |
| 4 | GO:0009941 | CC | chloroplast envelope | 0.000276102 | 0.025167492 |
| 4 | GO:0009526 | CC | plastid envelope | 0.000276102 | 0.025167492 |
| 3 | GO:0072329 | BP | monocarboxylic acid catabolic process | 0.000281002 | 0.025316301 |
| 5 | GO:1901700 | BP | response to oxygen-containing compound | 0.000290551 | 0.02587573 |
| 3 | GO:0009112 | BP | nucleobase metabolic process | 0.000410679 | 0.035459576 |
| 4 | GO:0031975 | CC | envelope | 0.000411895 | 0.035459576 |
| 4 | GO:0031967 | CC | organelle envelope | 0.000411895 | 0.035459576 |
| 7 | GO:0005739 | CC | mitochondrion | 0.000493001 | 0.041519229 |
| 6 | GO:0009628 | BP | response to abiotic stimulus | 0.00075631 | 0.063009561 |
| 32 | GO:0044237 | BP | cellular metabolic process | 0.000841707 | 0.069378127 |
| 4 | GO:0043413 | BP | macromolecule glycosylation | 0.001072676 | 0.083950428 |
| 4 | GO:0006486 | BP | protein glycosylation | 0.001072676 | 0.083950428 |
| 2 | GO:0016646 | MF | oxidoreductase activity, acting on the CH-NH group of donors, NAD or NADP as acceptor | 0.00105477 | 0.083950428 |
| 4 | GO:0070085 | BP | glycosylation | 0.001262444 | 0.09781419 |
| 1 | GO:0019285 | BP | glycine betaine biosynthetic process from choline | 0.001443212 | 0.100738821 |
| 1 | GO:0006577 | BP | amino-acid betaine metabolic process | 0.001443212 | 0.100738821 |
| 1 | GO:0006578 | BP | amino-acid betaine biosynthetic process | 0.001443212 | 0.100738821 |
| 1 | GO:0019695 | BP | choline metabolic process | 0.001443212 | 0.100738821 |
| 1 | GO:0009943 | BP | adaxial/abaxial axis specification | 0.001443212 | 0.100738821 |
| 1 | GO:0031455 | BP | glycine betaine metabolic process | 0.001443212 | 0.100738821 |
| 1 | GO:0031456 | BP | glycine betaine biosynthetic process | 0.001443212 | 0.100738821 |
| 1 | GO:0006540 | BP | glutamate decarboxylation to succinate | 0.001443212 | 0.100738821 |
| 1 | GO:0009798 | BP | axis specification | 0.001443212 | 0.100738821 |
| 1 | GO:0010492 | BP | maintenance of shoot apical meristem identity | 0.001443212 | 0.100738821 |
| 1 | GO:0009516 | CC | leucoplast | 0.001443212 | 0.100738821 |
| 6 | GO:0042221 | BP | response to chemical | 0.001763787 | 0.121151016 |
| 3 | GO:0072527 | BP | pyrimidine-containing compound metabolic process | 0.001766916 | 0.121151016 |
| 12 | GO:0044281 | BP | small molecule metabolic process | 0.001968365 | 0.13261648 |
| 4 | GO:0009725 | BP | response to hormone | 0.00205048 | 0.136957908 |
| 2 | GO:0051017 | BP | actin filament bundle assembly | 0.00213141 | 0.139950546 |
| 2 | GO:0061572 | BP | actin filament bundle organization | 0.00213141 | 0.139950546 |
| 3 | GO:0043648 | BP | dicarboxylic acid metabolic process | 0.00237479 | 0.154620795 |
| 4 | GO:0009719 | BP | response to endogenous stimulus | 0.002549719 | 0.163266305 |
| 4 | GO:1901607 | BP | alpha-amino acid biosynthetic process | 0.002596729 | 0.164913545 |
| 1 | GO:1902074 | BP | response to salt | 0.002884368 | 0.175969168 |
| 1 | GO:0010074 | BP | maintenance of meristem identity | 0.002884368 | 0.175969168 |
| 3 | GO:0006575 | BP | cellular modified amino acid metabolic process | 0.003411967 | 0.206530625 |
| 8 | GO:0044248 | BP | cellular catabolic process | 0.004520687 | 0.269432959 |
| 2 | GO:0046653 | BP | tetrahydrofolate metabolic process | 0.004660594 | 0.275350898 |
| 5 | GO:0008092 | MF | cytoskeletal protein binding | 0.004691058 | 0.275350898 |
| 4 | GO:0008652 | BP | cellular amino acid biosynthetic process | 0.004747596 | 0.276574266 |
| 1 | GO:0019827 | BP | stem cell population maintenance | 0.005760522 | 0.32818031 |
| 1 | GO:0097164 | BP | ammonium ion metabolic process | 0.005760522 | 0.32818031 |
| 1 | GO:0098727 | BP | maintenance of cell number | 0.005760522 | 0.32818031 |
| 2 | GO:0006730 | BP | one-carbon metabolic process | 0.006337834 | 0.354013739 |
| 2 | GO:0016645 | MF | oxidoreductase activity, acting on the CH-NH group of donors | 0.006640355 | 0.365503698 |
| 2 | GO:0006760 | BP | folic acid-containing compound metabolic process | 0.006949337 | 0.376527718 |
| 1 | GO:0048825 | BP | cotyledon development | 0.007195525 | 0.384726485 |
| 32 | GO:0071704 | BP | organic substance metabolic process | 0.007199966 | 0.384726485 |
| 2 | GO:0042558 | BP | pteridine-containing compound metabolic process | 0.009649182 | 0.512067561 |
| 4 | GO:0010033 | BP | response to organic substance | 0.009736362 | 0.513179137 |
| 2 | GO:0051015 | MF | actin filament binding | 0.014863247 | 0.752967545 |
| 3 | GO:0003774 | MF | motor activity | 0.015212194 | 0.752967545 |
| 8 | GO:1901575 | BP | organic substance catabolic process | 0.017815576 | 0.862719283 |
| 1 | GO:0006538 | BP | glutamate catabolic process | 0.020018877 | 0.963392919 |
| 17 | GO:1901564 | BP | organonitrogen compound metabolic process | 0.026316056 | 1 |
| 2 | GO:0007015 | BP | actin filament organization | 0.030709608 | 1 |
| 1 | GO:0009065 | BP | glutamine family amino acid catabolic process | 0.034075306 | 1 |
| 4 | GO:0016053 | BP | organic acid biosynthetic process | 0.042734812 | 1 |
| 4 | GO:0046394 | BP | carboxylic acid biosynthetic process | 0.042734812 | 1 |
| 2 | GO:0097435 | BP | supramolecular fiber organization | 0.044526829 | 1 |
| 32 | GO:0008152 | BP | metabolic process | 0.048350496 | 1 |
| 4 | GO:1990234 | CC | transferase complex | 0.042449221 | 1 |
| 1 | GO:0048037 | MF | obsolete cofactor binding | 0.032678669 | 1 |
