# Supplementary material for: Genome-wide characterization of aldehyde dehydrogenase gene family members in groundnut (Arachis hypogaea) and the analysis under saline-alkali stress
Source: Front Plant Sci. 2023 Feb 16;14:1097001. doi: 10.3389/fpls.2023.1097001 (PMC9978533; doi:10.3389/fpls.2023.1097001)
Supplement: Supplementary Table 1 — The primers sequence of ALDH members in groundnut. [file DataSheet_1.zip › table/Table 5.DOCX]

| No. | Pathway ID | Description | P-value |
| --- | --- | --- | --- |
| 1 | map00903 | Limonene and pinene degradation | 4.43E-55 |
| 2 | map00330 | Arginine and proline metabolism | 1.02E-53 |
| 3 | map00410 | beta-Alanine metabolism | 2.52E-51 |
| 4 | map00340 | Histidine metabolism | 6.75E-48 |
| 5 | map00280 | Valine, leucine and isoleucine degradation | 1.10E-47 |
| 6 | map00310 | Lysine degradation | 8.06E-44 |
| 7 | map00380 | Tryptophan metabolism | 6.51E-40 |
| 8 | map00053 | Ascorbate and aldarate metabolism | 1.10E-39 |
| 9 | map00071 | Fatty acid degradation | 4.07E-39 |
| 10 | map00010 | Glycolysis / Gluconeogenesis | 3.08E-38 |
| 11 | map00561 | Glycerolipid metabolism | 8.50E-35 |
| 12 | map00620 | Pyruvate metabolism | 7.77E-32 |
| 13 | map00940 | Phenylpropanoid biosynthesis | 2.29E-11 |
| 14 | map00260 | Glycine, serine and threonine metabolism | 3.93E-07 |
| 15 | map00250 | Alanine, aspartate and glutamate metabolism | 4.94E-05 |
| 16 | map00640 | Propanoate metabolism | 0.000432832 |
| 17 | map00650 | Butanoate metabolism | 0.000816289 |
| 18 | map00030 | Pentose phosphate pathway | 0.000934937 |
| 19 | map00562 | Inositol phosphate metabolism | 0.002186538 |
| 20 | map00051 | Fructose and mannose metabolism | 0.009178102 |
| 21 | map00230 | Purine metabolism | 0.109405226 |
| 22 | map00500 | Starch and sucrose metabolism | 0.112353488 |

Table S5.
